# Exploration of the Protective Mechanism of Bax Removal against Ischemia Reperfusion Injury of Skin Flap through the p38 Mitogen-Activated Protein Kinase Pathway

**DOI:** 10.1155/2022/1175078

**Published:** 2022-10-17

**Authors:** Yapeng Wang, Yongwei Wu, Peng Wang, Junhao Luo, Yongjun Rui

**Affiliations:** Department of Orthopaedics, Wuxi No. 9 People's Hospital Affiliated to Soochow University, Wuxi 214000 Jiangsu, China

## Abstract

This research is aimed at exploring the influences of the Bax gene in the p38 mitogen-activated protein kinase (MAPK) pathway and its protective mechanism against ischemia-reperfusion injury (IRI) of skin flap. Forty male Sprague-Dawley (SD) rats were equally divided into the experimental group (Bax gene knockout rats) and control group. The dorsal flap model was prepared, and the survival rate of flap was observed after surgery. The rat flap tissue was cut and stained with hematoxylin-eosin (HE) and in situ terminal deoxynucleotidyl transferase-mediated dUTP nick end labeling (TUNEL). The distribution characteristics of p38MAPK and Bax were detected to evaluate the protective mechanism of Bax gene knockout on IRI of skin flap. After surgery, the survival rate of flaps in the experimental group (82.32%, 70.28%) was significantly higher than that in the control group (57.64%, 46.14%) (*P* < 0.05). The results of HE staining showed that on the 1^st^ day after surgery, compared with those in the control group, the skin flaps of the rats in the experimental group were arranged more neatly. The results of TUNEL staining showed that compared with that of the control group, the tissue structure of the skin flap of the experimental group was normal and only a few apoptotic cells appeared. In addition, compared with that in the control group (7.14, 4.25, 3.48, 2.18/6.46, 7.12, 4.86, and 2.44), the expression of Bax and p38 MAPK in the experimental group (0.96, 0.81, 0.76, 0.55/1.63, 1.33, 1.01, and 0.56) significantly decreased (*P* < 0.05). In short, after the Bax gene was knocked out, injury of the flap after ischemia-reperfusion was considerably improved, which may play a protective role on the IRI of the flap by affecting the p38MAPK pathway.

## 1. Introduction

With the improvement of living standards and changes in lifestyles, the number of people with skin damage is increasing. Flap transplantation is the most commonly used technique for skin damage repair and plastic reconstruction. It can help injured people to solve the problem of serious injury of a large area of the skin or soft tissue [1]. However, complications of flap ischemia-reperfusion injury often occur after flap transplantation technology, which seriously affects the therapeutic effect of surgery and brings a heavy burden to the patient's family and body [2, 3]. Flap ischemia-reperfusion injury refers to the fact that when the tissue or organ that had ischemia was reperfused, the blood support was restored after reperfusion and some tissues or organs suffered more serious damage. It is manifested as tissue structure destruction or functional metabolic disorder. Clinically, the phenomenon of such skin flap ischemia-reperfusion injury after tissue reperfusion is aggravated as skin flap ischemia-reperfusion injury [4, 5]. When the flap tissue is reperfused after ischemia, the area that has ischemia has not received sufficient blood perfusion and the damage is further extended and aggravated. During ischemia and reperfusion of the flap, potassium channels are closed on the vascular endothelium, resulting in the inability of potassium ions to flow out [6, 7]. The phenomenon of “calcium overload” caused by a large influx of calcium ions further causes vasoconstriction and spasm, and blood perfusion is reduced again [8]. When the flap is undergoing ischemia and reperfusion, the use of intracellular adenosine triphosphate is accelerated, resulting in the restriction of the ion transport process and the disturbance of the tissue environment [9].

Studies indicated that in addition to cell necrosis during the occurrence of flap IRI, there is also massive cell apoptosis, which has become an important cause of skin flap necrosis [10, 11]. Apoptosis is also called programmed cell death, which refers to the physiological process of active cell death under the stimulation of specific signals. In recent years, many studies have discovered in the development of flap ischemia and reinjury, a variety of apoptosis genes, which include B-cell lymphoma-2 (Bcl-2) gene family, P53, immediate early genes, and caspase, plays an important role in the process of cell apoptosis. The interaction of a large number of genes that control apoptosis forms a complex structural network, which synergistically induces the occurrence and development of apoptosis during ischemia-reperfusion [12–14]. The Bcl-2 associated x (Bax) gene is one of the members of the Bcl-2 family, and 21% amino acid homologous sequences exist between it and Bcl-2. The gene is widely distributed in various tissues and organs in the body, including the heart, brain, kidney, stomach, intestine, lymph nodes, and skeletal muscle, and it is a gene that promotes apoptosis [15, 16]. Bax gene expression does not directly induce apoptosis but exerts its ability to induce apoptosis through interaction with other proteins or signal pathways [17, 18]. In the mitogen-activated protein kinase (MAPK) family of mammalian cells, p38MAPK is one of the very important family members. After activation, it can play a variety of regulatory roles, including cell apoptosis, inflammation, and cell proliferation [19–22]. Therefore, in the study of flap ischemia and reinjury mechanism, the regulation of the p38MAPK signaling pathway and the role of the Bax gene in it have attracted extensive attention and research.

In this study, 40 male Sprague-Dawley (SD) rats were purchased from Wuxi No. 9 People's Hospital Affiliated to Soochow University, which were divided into the experimental group and control group with 20 rats in each group. The experimental group was Bax gene knockout rats, and the back flap model was prepared. The flap survival rate was observed after surgery. Rat skin flap tissue was cut, and hematoxylin-eosin (HE) staining and in situ terminal deoxynucleotidyl transferase-mediated dUTP nick end labeling (TUNEL) staining were performed. The distribution characteristics of p38MAPK and Bax were detected to investigate the effect of Bax gene knockout on the MAPK pathway and its protective mechanism on IRI of skin flap.

## 2. Materials and Methods

### 2.1. Experimental Animals

All SD rats used in this experiment were purchased from Wuxi No. 9 People's Hospital Affiliated to Soochow University, all male, weighing about 300 g, aged 49–56 days. There were 40 SD rats in total. All rats were divided into the experimental group and control group, with 20 rats in each group. The experimental group of rats was composed of Bax gene knockout rats and raised in a well-ventilated animal experimental base. During the experiment, all treatments of SD rats were strictly carried out in accordance with the national experimental animal regulations. This animal experiment had been approved by the ethical committee of laboratory animals.

### 2.2. Main Reagent

Hematoxylin dye solution, eosin dye solution, and TUNEL reaction solution were purchased from Shanghai Yuanye Biotechnology Co. Ltd. Pentobarbital sodium and DAB chromogenic kits were purchased from Changzhou Lier Chemical Co. Ltd. The reverse transcription kit was purchased from Shanghai Qiyuan Biotechnology Co. Ltd. PCR reagents were purchased from Shanghai Fantai Biotechnology Co. Ltd.

### 2.3. Establishment of a Model of IRI in Rat Back Flap

SD rats in the experimental group and control group were fixed and anesthetized by intraperitoneal injection of 2% pentobarbital sodium at a standard dosage of 30 mg/mL. After the rats were anesthetized, the back hair was removed, rectangular flaps of 7.5 × 2.0 cm were marked, and routine disinfection was performed twice with iodophor. The disposable sterile sheet was paved in the surgical scope, and the skin and subcutaneous tissue were cut according to the marked area. Then, the subcutaneous tissue was separated and the vascular pedicle was clipped for 6 h. After that, the vascular clamp was removed, and the blood flow was restored. The flap was sutured in situ with a 3-0 suture line. The principle of surgical aseptic operation should be strictly observed throughout the operation.

### 2.4. Measurement of the Survival Rate of Postoperative Skin Flap in Rats

The morphological characteristics of back flap tissue of the experimental group and control group were observed on the 1st, 3rd, and 7th day after operation. The color, swelling, wound healing, hair growth, and texture of the skin flap were observed. Flap images of each period were collected. Then, the survival rate of the skin flap was calculated according to the observed image characteristics and the criteria of skin necrosis, including black skin, crusted skin on the skin surface, loss of elasticity, and hardness of the skin flap texture. In addition, the total area of the flap was denoted by *S*, the necrosis area of the flap was denoted by *D*, and the survival rate of the flap was denoted by *R*. The survival rate of the rat flap in each group was calculated according to the following:

(1) $$\begin{array}{c} R=\frac{S-D}{S}\times 100\%. \end{array}$$

### 2.5. Staining of Rat Skin Flap Specimens

HE staining SD rat flaps were sacrificed at 1 h, 1 d, 3 d, and 7 d after operation. The skin of the injured part of the rat flap was treated with formalin, and the paraffin-embedded specimens were cut into 5 *μ*m thick sections in a slicer for HE staining: paraffin sections were successively treated with hematoxylin, water, alcohol hydrochloric acid, water, ammonia, water, and eosin. Alcohol gradient dehydration was carried out, and cleaning and xylene transparent were performed before pictures were taken under a microscope observation.

TUNEL staining SD rat flaps were sacrificed at 1 h, 1 d, 3 d, and 7 d after operation. The injured skin of rat skin flap was treated in formalin. The paraffin-embedded specimens were cut into 5 *μ*m thick sections in the slicer for TUNEL staining. Paraffin sections were treated with xylene, ethanol, water, trypsin K, PBS, and TUNEL reaction solution. They were put into a wet box to ensure a certain humidity environment, incubated at 37°C for 1 h, and washed with PBS for 3 times. Then, they were stained with DAB stain for 30 min, washed with PBS for 3 times, and put into hematoxylin staining solution for 10 min. Alcohol gradient dehydration was carried out, and cleaning and xylene transparent were performed before pictures were taken under a microscope observation.

### 2.6. Determination of the Content of Bax and p38MAPK in Skin Flaps

At 1 h, 1 d, 3 d, and 7 d after SD rat flap surgery, 3 rats in each group were sacrificed and part of the damaged tissue was first put into normal saline for full rinsing. Then, total RNA was extracted from the flap tissue, and quantitative real-time polymerase chain reaction (qRT-PCR) was implemented to detect the content of Bax and p38MAPK in the flap tissue. The reference gene was PDH, and the result calculation equation was *F* = 2^−△△*Ct*^. PCR primers were provided by the XXX Company, and the primer sequences were shown in Table 1 as follows.

### 2.7. Statistical Methods

SPSS was used for statistical analysis of data, and the data conforming to normal distribution was expressed as mean plus or minus standard deviation (mean ± SD). *t*-Test was used to represent measurement data, chi-squared (*χ*^2^) test was used to represent count data, and *P* < 0.05 indicated statistical difference.

## 3. Results

### 3.1. Observation Results of the Flap Survival Rate and Morphology

**Figure 1:**
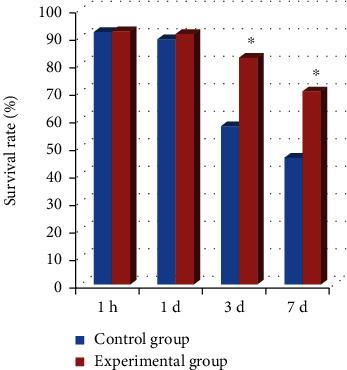
Results of the survival rate of the two groups of rat skin flaps. ^∗^Remarkable difference, *P* < 0.05.

At 1 h, 1 d, 3 d, and 7 d after operation, the morphological characteristics of the back flap tissue of experimental group and control group were observed to calculate the survival rate of the flap. The results showed that there was no remarkable difference in the flap survival rate between the experimental group and the control group at 1 h and 1 d after surgery (*P* > 0.05). The survival rate of skin flap in the experimental group was greatly higher than that in the control group at 3 d and 7 d after surgery (*P* < 0.05) (Figure 1).

**Figure 2:**
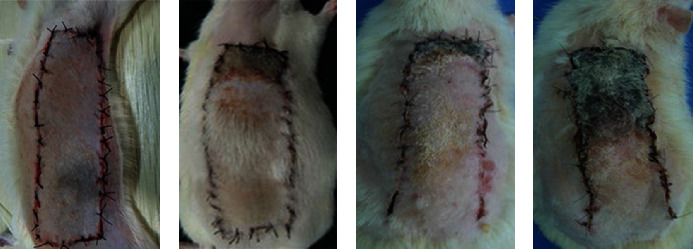
Morphological observation of postoperative skin flaps in the two groups of rats. (a, b) The tissue morphology of rat skin flaps in the test group and control group 3 days after operation, respectively; (c, d) the tissue morphology of rat skin flaps in the test group and control group 7 days after operation, respectively.

The morphological changes of the skin flaps of the two groups were observed after operation. The results showed that the rats could eat normally three days after surgery, and there was no obvious necrosis at the edge of the skin flap in the experimental group. The skin flap tissue was elastic and no black scab was formed. A small amount of necrotic flap tissue was found in the control group. The two groups of rats were relatively active and in good condition 7 d after surgery. A small amount of necrotic tissue appeared in the experimental group, and skin texture did not change considerably. Rats in the control group showed a large number of necrotic scabs, and the flap tissue became hard and lost elasticity. The specific results were shown in Figure 2.

### 3.2. HE Staining Morphology Results

**Figure 3:**
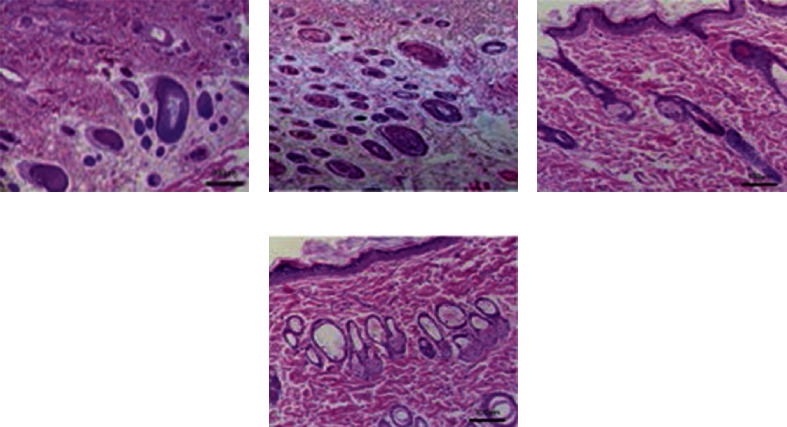
HE staining results of postoperative skin flaps of two groups of rats. (a, b) The HE staining of rat skin flaps in the control group 3 and 7 days after operation, respectively; (c, d) the HE staining of rat skin flaps in the experimental group 3 and 7 days after operation, respectively.

The two groups of rats were observed by HE staining of skin flap tissue after surgery, and the results showed that three days after surgery, the skin flap tissue of rats in the experimental group was orderly arranged with a small number of inflammatory cells. In contrast, there were more inflammatory cells in the control group and their arrangement was chaotic. On day seven after surgery, cells of the experimental group were orderly arranged and a few inflammatory cells still appeared. In contrast, cells in the control group were disordered, with massive infiltrated inflammatory cells, and sparse nuclei were observed (Figure 3).

### 3.3. TUNEL Staining Morphological Results

**Figure 4:**
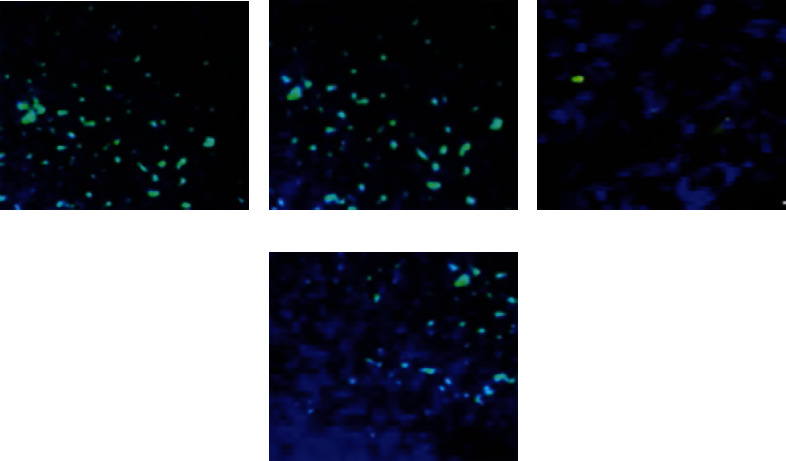
TUNEL staining results of postoperative skin flaps in two groups of rats. Blue was the nuclear staining of viable cells, and green was the nuclear staining of postapoptotic cells.

TUNEL staining was performed on the rat flaps in each group, and the surviving nucleus was blue, while the nucleus after apoptosis was green. Three days after operation, the cell structure and morphology of the flaps in the experimental group were normal, while a few apoptotic cells appeared in the control group. On the 7th day after surgery, the tissue structure of the flaps in the experimental group was relatively intact with a small amount of apoptosis, while the cells in the control group were largely apoptotic and the tissue structure of the flaps was abnormal. The specific results were shown in Figure 4. TUNEL staining was performed on the rat flaps in each group, and the surviving nucleus was blue, while the nucleus after apoptosis was green. Three days after operation, the cell structure and morphology of the flaps in the experimental group were normal, while a few apoptotic cells appeared in the control group. On the 7th day after surgery, the tissue structure of the flaps in the experimental group was relatively intact with a small amount of apoptosis, while the cells in the control group were largely apoptotic and the tissue structure of the flaps was abnormal. The specific results were shown in Figure 4.

Figures 4(a) and 4(b) showed the TUNEL staining of rat skin flaps in the control group at 3 and 7 days after operation, respectively; Figures 4(c) and 4(d) showed the TUNEL staining of rat skin flaps in the experimental group 3 d and 7 d after operation, respectively.

### 3.4. Test Results of the Bax Content in Skin Flaps

**Figure 5:**
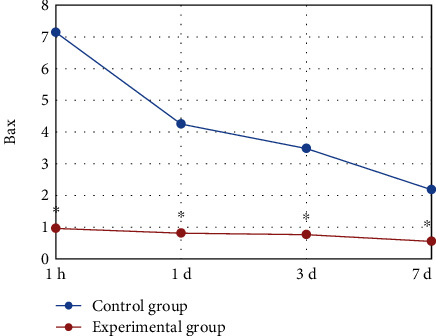
The results of the change trend of the Bax content in the skin flap tissues of the two groups of rats after surgery. ^∗^ indicated that compared with the control group, the difference was statistically significant (*P* < 0.05).

At 1 h, 1, 3, and 7 days after the surgery, the Bax content in the flap tissue was detected. Compared with that of the control group, the Bax content in the skin flap tissue of the experimental group was significantly decreased (*P* < 0.05). The Bax content of the Bax knockout rats in the group was at a low level after the surgery, while that of the control group rats gradually decreased. The specific results were shown in Figure 5.

### 3.5. Test Results of p38MAPK Content in Skin Flaps

**Figure 6:**
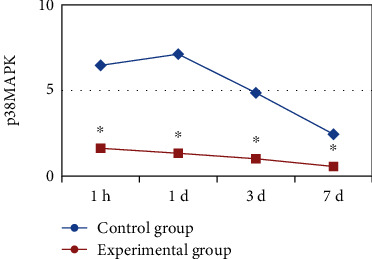
Change trend of the p38MAPK content in the skin flap tissues of the two groups of rats after surgery. ^∗^ indicated that compared with the control group, the difference was statistically significant (*P* < 0.05).

At 1 h, 1, 3, and 7 days after the surgery, the content of p38MAPK in the flap tissue was detected. There was a significant difference in the p38MAPK content between the two groups of rats (*P* < 0.05). The p38MAPK content in the middle group was lower than that in the control group, and that of the Bax gene knockout rats in the experimental group was always at a low level after the operation, while that in the control group showed a trend of gradually decreasing. The specific results were shown in Figure 6.

## 4. Discussion

With the rapid development of the society and the diversification of lifestyles, there are more and more open clinical injuries, usually manifested as large-scale skin injuries. In severe cases, tendons and bones are exposed and complications such as infection and osteomyelitis are easily caused, which brings great pressure to clinical treatment. The skin flap transplantation technology treats and repairs such skin injuries and completes plastic reconstruction. At present, the technology has matured and has become an important technical means and is widely used in the treatment of large-area skin or soft tissue serious injuries [23, 24]. However, during the application of flap transplantation technology, flap IRI often occurs, which is the most common complication after flap transplantation. It severely affected the therapeutic effect of surgery and brought a heavy burden to the patient's family and body [25]. The long-term ischemia of the flap tissue leads to ischemic injury. When the blood perfusion is restored, the injury of the flap is aggravated and it even causes the necrosis of the flap tissue, which has a huge negative impact on the success rate of the flap transplantation. However, due to the complexity of the injury process itself, the specific mechanism of this injury is still unclear. Therefore, it is particularly important to continue to study the specific mechanism of flap IRI and its mitigation methods [26]. Animal models commonly used to study flap IRI include pigs, rabbits, and mice. The application of the rat skin flap model is the most common and extensive. There are two kinds of rat skin flap models, that are, the classical rat abdominal skin flap model and rat back skin flap model. The abdominal flap model has helped researchers achieve effective research results in alleviating flap IRI. However, the flap tissue is often damaged by rats' eating in the practical application of the rat flap model, resulting in serious infection, which has a serious impact on the final test results. The rat back model can overcome such limitations [27, 28]. In this study, 40 male SD rats were purchased and divided into the experimental group and control group, with 20 in each group. Rats in the experimental group were Bax gene knockout rats, and back flap models were prepared in both groups to explore the mechanism of flap IRI.

In clinic, prevention or cure of flap IRI is the common pursuit of doctors and researchers. The p38MAPK pathway has been found to play a key role in studies on the mechanisms related to IRI of flaps [29]. In mammalian cells, the p38MAPK signaling pathway plays an important role in many aspects, including regulation of inflammation, cell proliferation, and apoptosis [30]. The inhibition of p38MAPK can effectively limit the secretion of proinflammatory cytokines, alleviate the damage caused by inflammation, and ultimately improve the situation of IRI of skin flap. Moreover, it can also regulate the apoptosis process of skin flap cells and relieve the necrosis of skin flap [31]. The Bax gene is a gene that promotes apoptosis, and its expression does not directly induce apoptosis but exerts its ability to induce apoptosis through interaction with other proteins or signaling pathways [32]. In this study, after fabrication of the back flap model of the experimental group and control group, the survival rate of the flap was observed after surgery. Rat skin flap tissue was cut for HE staining and TUNEL staining. The distribution characteristics of p38MAPK and Bax were detected to evaluate the protective mechanism of Bax gene knockout on IRI of skin flap. The results showed that there was no remarkable difference in the flap survival rate between the experimental group and the control group at 1 h and 1 d after surgery (*P* > 0.05), respectively. The survival rate of the skin flap in the experimental group was considerably higher than that in the control group at 3 d and 7 d after surgery (*P* < 0.05), indicating that the IRI of the skin flap in the experimental group was improved after Bax gene knockout. HE and TUNEL staining showed that the cells of the experimental group were orderly arranged, and a small number of inflammatory cells appeared on the 7th day after surgery. The control rats, on the contrary, had a chaotic array of infiltrating inflammatory cells. At the same time, sparse nucleus and a large number of apoptotic cells were observed, indicating that Bax knockout can alleviate the injury of the rat skin flap tissue in the model of ischemia-reperfusion, and had a certain preventive effect. In addition, compared with that in the control group, the expression of Bax and p38MAPK in experimental group was considerably decreased (*P* < 0.05). The variation trend of Bax and p38MAPK contents in the experimental group was relatively uniform, indicating that the knockout of Bax gene affected the p38MAPK pathway and thus played a protective role in the IRI of the skin flap. Some studies suggested that the degree of IRI of flaps was reduced after the p38MARK inhibitor was used to reduce the content of p38MAPK, which was consistent with this study [33].

## 5. Conclusion

In this work, the effect of Bax gene knockout on the 38MAPK pathway and its protective mechanism on flap ischemia-reperfusion injury were investigated in rats in the control group and experimental group. The results showed that knockout of the Bax gene improved the injury of rat skin flap in the model of skin flap ischemia-reperfusion and had a certain preventive effect and may play a protective role against skin flap ischemia-reperfusion injury by affecting the p38MAPK pathway. The disadvantage was that the grouping was relatively single and there are few control variables. In the next study, a p38MAPK inhibitor group can be set to compare the improvement of skin flap injury in each group and to further explore and study the mechanism of skin flap ischemia-reperfusion injury. It provided a more practical and effective reference value for the treatment and prevention of flap injury.

## Figures and Tables

**Table 1:** Primer sequences.

Primer	Sequence (5′⟶3′)
Bax-F	CGGAATTCATGGACGGGTCCGGGGAG
Bax-R	CCGCTCGAGTCAGCCCATCTTCTTCCAG
p38MAPK-F	ATGCCATTACCAGTCTCCG
p38MAPK-R	TGCAGCATCAATACGGTTG
GAPDH-F	GATGCTGGTGCTGAGTATGRCG
GAPDH-R	GTGGTGCAGGATGCATTGCTCTGA

## Data Availability

All data, models, and code generated or used during the study appear in the submitted article.
